# Simple Models of Complex Mechanics for Improved Hypertension Care: Learning to De-stiffen Arteries

**DOI:** 10.1007/s44200-023-00037-1

**Published:** 2023-07-25

**Authors:** Ryan J. Pewowaruk

**Affiliations:** 1 Research Service, William S. Middleton Memorial Veterans Hospital, Madison, WI USA; 2 Department of Medicine Division of Cardiovascular Medicine, University of WI – Madison, Madison, WI USA

**Keywords:** Vascular stiffness, Extracellular matrix, Vascular smooth muscle cells, Blood pressure

## Abstract

Arteries can stiffen via different mechanisms due to the distending effects of blood pressure, the extracellular (ECM) and vascular smooth muscle cells (VSMC). This short review discusses how these simple models can be applied to the complex biomechanics of arteries to gain physiological insight into why an individual’s arteries are stiff and identify new therapeutic strategies. In the Multi-Ethnic Study of Atherosclerosis, the important question of whether arteries stiffen with aging due to load-dependent or structural stiffening was investigated. Structural stiffening was consistently observed with aging, but load-dependent stiffening was highly variable. Importantly, the high load-dependent stiffness was associated with future cardiovascular disease events, but structural stiffness was not. Clinical studies in older, hypertensive adults surprisingly show that decreasing vascular smooth muscle tone can cause clinically significant increases in arterial stiffness. To understand this paradox, the author developed a model simple enough for clinical data but with biologically relevant extracellular matrix (ECM) and vascular smooth muscle cell (VSMC) stiffness parameters. The effect of VSMC tone on arterial stiffness depends on the ECM–VSMC stiffness ratio. Future research is needed to develop a framework that incorporates both the blood pressure dependence of arterial stiffness and the VSMC–ECM interaction on hemodynamics. This could result in personalized arterial stiffness treatments and improved CVD outcomes. The subtitle of this review is “Learning to De-Stiffen Arteries” because our results have so far only shown that we can acutely make arteries stiffer. We are optimistic though that the findings and the analytic techniques covered here will be one of the many steps along the path of the arterial stiffness research community learning how to de-stiffen arteries.

## Introduction

Hypertension is a major cause of renal, neural, and cardiovascular disease (CVD), putting the many individuals with poor blood pressure control at high risk for end-organ damage and death [1]. Arterial stiffness, or the rigidity of the arterial wall, increases with hypertension, aging, and other common conditions [2] and is associated with future CVD events [3]. The SPARTE trial showed that an arterial stiffness-based approach to antihypertensive therapy improved blood pressure and limited arterial stiffening with aging but did not show a significant benefit with regard to cardiovascular disease events [4]. Hypertension is treatable but guideline statements are inconsistent with regard to including arterial stiffness measures in hypertension treatment algorithms. The inconsistency of guideline statements is at least in part due to a lack of results from clinical trials that performing arterial stiffness measurements will improve clinical outcomes. There is great potential for personalizing hypertensive care based on arterial stiffness if it could be shown that stiffness-focused interventions can lower CVD risk. There is a critical need for proven, patient-specific approaches to de-stiffen arteries in hypertension.

Arteries can stiffen via different mechanisms and without understanding why an individual’s arteries are stiff, the ability to personalize treatment based on arterial stiffness is limited. Mathematical models of arterial biomechanics are a useful tool for understanding the blood pressure dependence of arterial stiffness in addition to factors such as extracellular matrix (ECM) vs. vascular smooth muscle cell (VSMC) mechanics [5, 6]. However, arteries have multiple layers, multiple constituents, and a non-linear response to blood pressure so biomechanics models of arteries are typically too complex to be used with the limited data that can be measured in clinical studies [7]. Simple models based on pressure–diameter measurements have utility though in that they can be applied to data collected in humans. Models with a single exponential form are particularly advantageous because they adequately capture the non-linear behavior of arteries but only require measurement of systolic and diastolic pressure–diameter data points, or a single pulse wave velocity and pressure data point. In fact, the first model proposed by Y.C. Fung in 1967 for non-linear soft tissue mechanics was a single exponential model [8]. These simple models of arterial stiffness have continued to be used for nearly 60 years since then in work such as that of Hayashi in the 1970s [9], Kawasaki in the 1980s [10], Lehman in the 1990s [11], and Spronck in the 2010s [12].

This short review discusses how these simple models can be applied to the complex biomechanics of arteries to gain physiological insight into why an individual’s arteries are stiff and identify new therapeutic strategies. The subtitle of this review is “Learning to De-Stiffen Arteries” because we have so far only shown that we can acutely make arteries stiffer. We are optimistic though that the findings and the analytic techniques covered here will be one of the many steps along the path of the arterial stiffness research community learning how to de-stiffen arteries.

## Blood Pressure Dependence of Arterial Stiffness

**Fig. 1:**
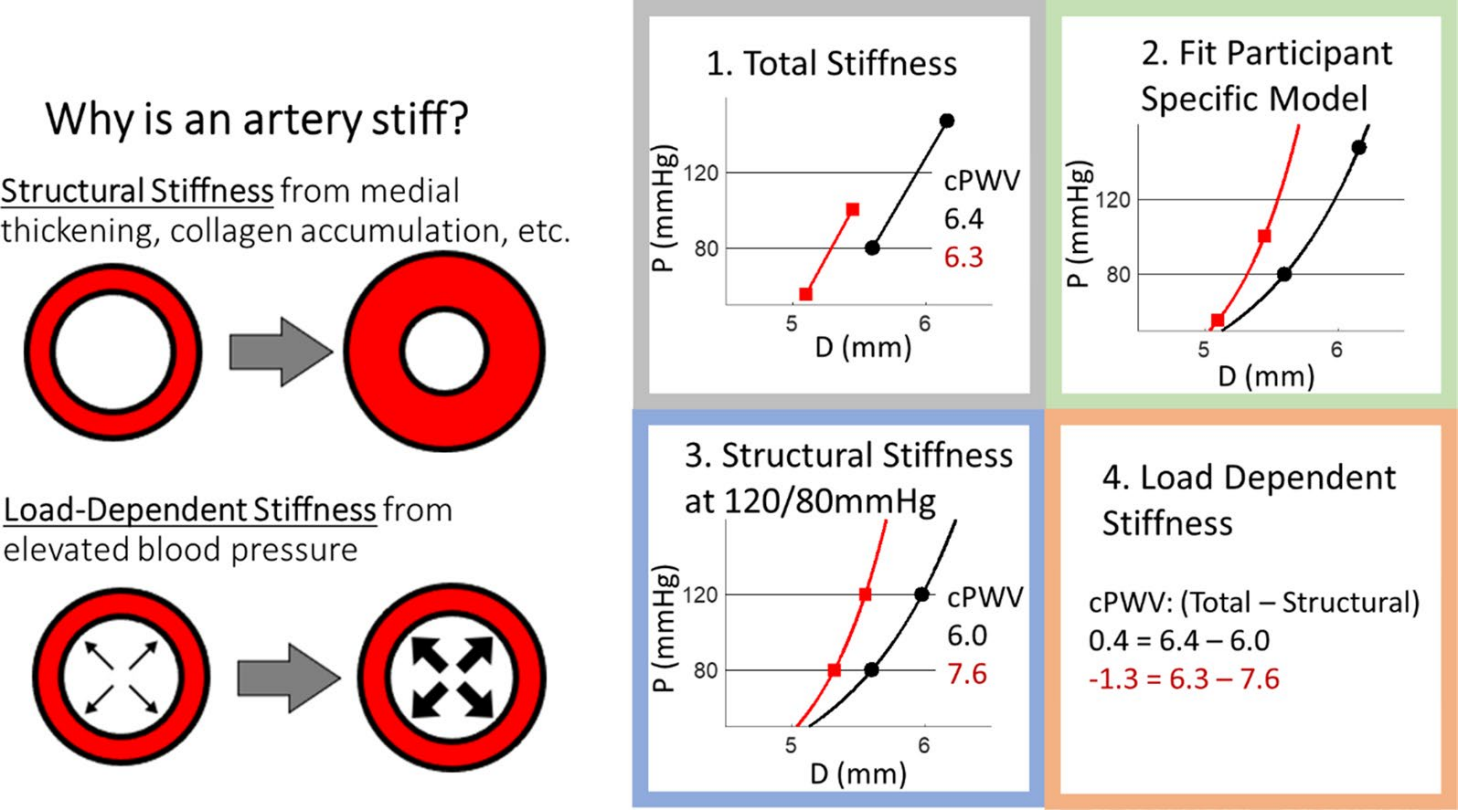
Graphical representation of methods used to differentiate structural and load-dependent stiffness [14, 15]. Representative results are shown for two participants (one in red, one in black) who had similar total carotid pulse wave velocity (cPWV) (6.4 vs 6.3 m/s), but via different mechanisms. One participant (red lines and text) had higher structural cPWV (7.6 vs 6.0 mmHg) while the other participant (black lines and font) had higher load-dependent cPWV (0.4 vs − 1.3 mmHg)

Using carotid artery ultrasound data from the Multi-Ethnic Study (MESA) [13], myself and colleagues endeavored to answer the important question in vascular aging of whether arteries stiffen with aging due to (1) load-dependent stiffening from higher blood pressure acutely recruiting collagen fibers as the artery distends, or (2) structural stiffening due to remodeling of the vessel wall [14]. To differentiate these two mechanisms, we used single exponential models of artery pressure–diameter relationships (Fig. 1),

1 $$\begin{array}{c}P={P}_{d}{e}^{\gamma \left(\frac{{D}^{2}}{{D}_{d}^{2}}-1\right)}\end{array}$$

where $\gamma$ is the non-linear stiffness parameter calculated

2 $$\begin{array}{c}\gamma =\frac{\mathrm{ln}\left(\frac{{P}_{s}}{{P}_{d}}\right)}{\frac{{D}_{s}^{2}}{{D}_{d}^{2}}-1}\end{array}$$

First, a traditional stiffness metric (e.g., pulse wave velocity, Peterson’s elastic modulus, Young’s elastic modulus) is calculated at the individual’s actual blood pressure to represent total arterial stiffness. Second, a single exponential model is fitted to the measured pressure–diameter data using Eq. 2. In the case of only measuring systolic and diastolic data points, the model uniquely and exactly fits these two points. Third, structural stiffness is calculated at the same 120/80 mmHg blood pressure for all individuals using participant-specific models. For example, Eq. 1 can be inserted in the Bramwell–Hill equation to calculate pulse wave velocity at a 120/80 mmHg blood pressure

3 $$\begin{array}{c}PW{V}_{120/80}=\sqrt{\frac{120-80}{\mathrm{ln}\left(\frac{120}{80}\right)}\left(\gamma +\mathrm{ln}\left(\frac{80}{{P}_{d}}\right)\right)}\end{array}$$

Last, load-dependent stiffness is calculated as the difference between total stiffness and structural stiffness. For example, load-dependent pulse wave velocity (*PWV*_*LD*_) would be calculated

4 $$\begin{array}{c}PW{V}_{LD}=PWV-PW{V}_{120/80}\end{array}$$

**Fig. 2:**
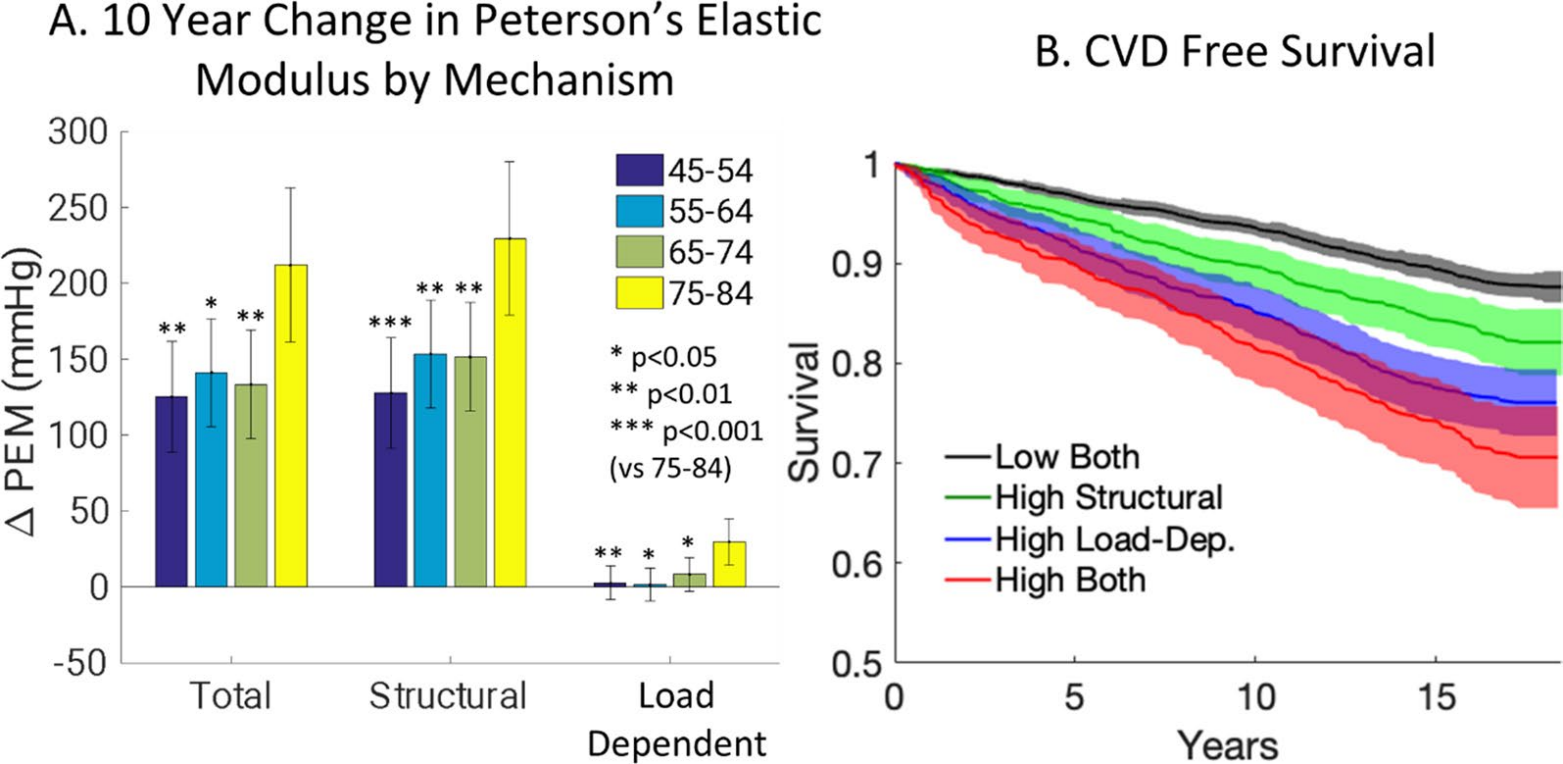
**A** Estimated means and 95% CIs for total, structural, and load-dependent changes in carotid artery Peterson elastic modulus (PEM) over 10 years of aging by age groups in *n* = 2604 MESA participants. Reproduced from [14].** B** Unadjusted Kaplan–Meier curves (with 95% CIs) show that cardiovascular disease (CVD)-free survival is greater for MESA participants (*n* = 5873) with high structural vs high load-dependent stiffness. Reproduced from [15]

Over a decade of follow-up with carotid ultrasound in MESA (*n* = 2604 adults at 6 sites in the United States, ages 45–84 at baseline), we found that structural stiffening was consistently observed with aging but that load-dependent stiffening was highly variable (Fig. 2) [14]. Load-dependent stiffness was significantly associated in ANCOVA models with blood pressure as expected, but also with risk factors such as cholesterol, race/ethnicity, and socio-economic status. Changes in structural stiffness were primarily associated with age and not modifiable risk factors. On average, approximately 85–100% of the stiffness increases with aging were due to structural stiffening depending on participants’ age, but there was high heterogeneity with 1/3rd of participants having greater load-dependent stiffening vs. structural stiffening.

As a follow-up analysis, we looked at the association between load-dependent vs structural stiffening with CVD events and incident hypertension in MESA in 5873 participants over an average of 14.3 year of participant follow-up [15]. Surprisingly, we found that only load-dependent stiffness was associated with CVD events and incident hypertension, but that structural stiffness was not (Fig. 2). An additional important finding for researchers using arterial stiffness measurements was that total arterial stiffness was associated with CVD events after adjusting for blood pressure with a traditional statistical model (Cox proportional hazard analysis), but structural stiffness which was adjusted for blood pressure using participant-specific models was not associated with CVD events. We also examined the association of structural and load-dependent stiffness with all-cause mortality, chronic kidney disease (CKD), and dementia in MESA (*n* = 6147, average 14.3 years follow-up) [16]. Structural stiffness was associated with all‐cause mortality, and load‐dependent stiffness was associated with CKD. Total stiffness was associated with dementia, but load‐dependent and structural stiffness were not. Taken together, these analyses in MESA emphasize the importance of BP control to reduce the impact of arterial stiffness in CVD and CKD and highlight the need to develop approaches to improve arterial stiffness independent of lowering BP to reduce all‐cause mortality. It is unclear why structural stiffness is associated with all‐cause mortality but not CKD, dementia, or CVD, and this requires further study.

## Vascular Smooth Muscle Cells in Arterial Stiffness

Historically, increased large elastic artery stiffness has been believed to be primarily due to two main extracellular matrix (ECM)-related mechanisms: (1) load-dependent stiffening due to higher blood pressure and (2) structural stiffening representative of changes intrinsic to the artery. However, there is an increasing amount of data showing that arterial stiffness can acutely be modified independently of blood pressure in humans [17, 18], which suggests an important role of vascular smooth muscle cells (VSMCs), in addition to the known importance of the ECM. Adding to this literature, our group’s ongoing studies in older adults (> 60 years old) show that acute reductions in VSMC tone with nitroglycerin unexpectedly cause large and clinically significant increases in elastic artery stiffness of hypertensive Veterans [19]. To better understand why relaxing VSMCs paradoxically makes arteries stiffer in older adults, the author developed a novel arterial mechanics model based on pressure–diameter relationships that incorporates the contributions of ECM and VSMC to arterial stiffness measures [20]:

5 $$\begin{array}{c}P={P}_{ref}\left({e}^{{\gamma }_{ECM}\left(\frac{D}{{D}_{ref}}-1\right)}+\frac{k}{{k}_{ref}}{e}^{{\gamma }_{VSMC}\left(\frac{D}{\left(1-k\right){D}_{ref}}-1\right)}\right)\end{array}$$

where ${P}_{ref}$ is a reference pressure, ${\gamma }_{ECM}$ is the ECM stiffness, *k* is the VSMC activation (*k* = 0 equates to fully relaxed VSMCs), *k*_*ref*_ is a reference VSMC activation, and ${\gamma }_{VSMC}$ is the VSMC stiffness. This model is advantageous because it is simple enough to use with limited clinical data but has biologically relevant parameters which include ECM stiffness, smooth muscle stiffness, and smooth muscle tone. Despite its simplicity, the model is able to accurately represent brachial artery mechanics over a wide range of blood pressures and vascular smooth muscle tones [20]. After using the model to retrospectively analyze several clinical studies, there appears to be a simple explanation for the role of smooth muscle contraction on arterial stiffness: Increased VSMC contraction increases arterial stiffness when VSMC is stiffer than ECM and decreases arterial stiffness when ECM is stiffer than VSMC.

**Fig. 3:**
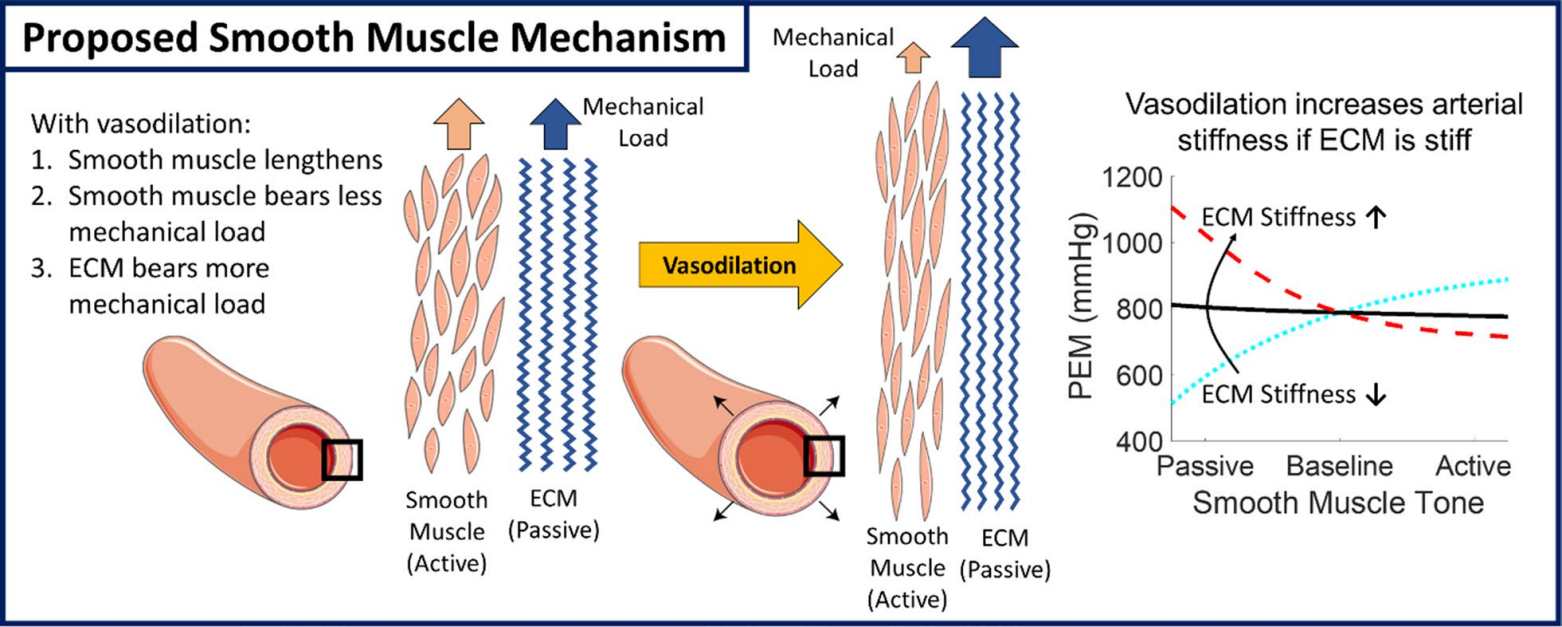
Proposed mechanism for elastic artery stiffening with vasodilation in older adults. With vasodilation, decreasing smooth muscle tone, vascular smooth muscle supports less mechanical load and the ECM supports more mechanical load. If the ECM is stiffer than the smooth muscle (as is likely the case in older adults), vasodilation shifting load from smooth muscle to the ECM will make the artery stiffer. *PEM* Peterson’s elastic modulus. Parts of the figure were created with images from Servier Medical Art. Servier Medical Art by Servier is licensed under a Creative Commons Attribution 3.0 Unported License. Figure from [19]

This work has been highlighted in editorials as an important first step toward understanding the role of VSMCs in arterial dysfunction [21] and raising the possibility of adverse effects of vasodilatory medications in older hypertensive individuals [22]. Based on our data and analysis, we hypothesize a framework where active vascular smooth muscle cell and passive ECM mechanics interact to determine elastic artery stiffness (Fig. 3). This framework has important implications for novel therapeutic strategies in hypertension. Understanding how smooth muscle contributes to large artery stiffness could be used for improved CVD risk stratification and to personalize the threshold for initiating hypertensive care and medication selection. Supporting this idea, in individuals whom nitroglycerin increases carotid artery stiffness, prevalence and severity of coronary artery disease are greater compared to those in whom nitroglycerin decreased carotid artery stiffness [23]. There is also potential to reduce arterial stiffness through changes in smooth muscle tone with medications. We explored this potential therapeutic benefit with participant-specific simulations of carotid artery biomechanics in the hypertensive Veterans who had arterial stiffness measured before and after nitroglycerin administration. Simulations predicted that *increasing* smooth muscle tone can have a stronger or equivalent effect on improving carotid artery mechanics compared with decreasing blood pressure [24], although these simulations require future experimental validation.

VSMC mechanics may also contribute to hypertensive response to exercise (excessively increased blood pressure with exercise), which is associated with left ventricular hypertrophy and higher rates of CVD [25]. Exercise data from the same older participants as our nitroglycerin study shows that a maximal treadmill stress test elevates cf-PWV independently of increased blood pressure [26]. Theoretically, higher arterial stiffness would lead to higher pulse pressures, and experimentally we found that after correcting for diastolic blood pressure with an exponential model (because foot-foot cf-PWV is diastolic stiffness), higher post-exercise cf-PWV was correlated with higher post-exercise systolic blood pressure. This finding highlights the potentially adverse effects of increased arterial stiffness with exercise on hemodynamics and ultimately the development of cardiac dysfunction.

## Future Directions

The cellular and molecular mechanisms underlying changes in the blood pressure dependence of stiffness and the interplay of VSMC and ECM on arterial stiffness are undoubtedly important and should be a focus of future research. It will be equally important, however, to integrate the effect of these changes in central and conduit artery stiffness into the hemodynamics of the whole cardiovascular system. Central–peripheral arterial interactions can be quantified through wave reflections using either time or frequency domain approaches [27]. Wave reflection magnitude (*R*_m_) is associated with incident heart failure events [28]. From 1D fluid mechanics, wave reflections occur due to a mismatch of proximal–distal impedance (*Z*) in the circulation [29]. Impedance is calculated as follows: $Z=\rho \frac{PWV}{A}$, where $\rho$ is the density of blood, *PWV* is pulse wave velocity, and *A* is artery cross-sectional area. In each individual, *R*_m_ could be elevated due to remodeling of the central arteries, remodeling of the peripheral arteries, the non-linear effect of elevated blood pressure changing arterial stiffness and thus impedance, or a combination of these effects. Simple 1D models of hemodynamics, such as a T-tube model [30] for the systemic circulation, a structured tree model [31] for the pulmonary circulation, or distributed lumped parameter models [32] could be modified from their linear arterial stiffness to an exponential model of arterial stiffness. These models could then be applied to experimental data to help differentiate the load-dependent vs. structural stiffening mechanisms that could contribute to altered macro–micro vascular interactions.

It may also be possible to understand the impact of VSMC–ECM interactions on central and peripheral arterial mechanics by measuring pressure-flow relationships before and after administration of a vasoactive agent. This analysis could be performed by incorporating the simple exponential smooth muscle mechanics model as a constitutive relationship into 1D hemodynamics models. This analytical approach to assess VSMC–ECM mechanical interactions in the central vs. peripheral vasculature holds great promise for holistically understanding circulatory physiology but would be dependent on future research identifying what experimental data need to be collected to uniquely identify all parameters and if this data could be collected non-invasively in humans.

## Conclusion

Arteries can stiffen via different mechanisms and simple models can be applied to complex arterial mechanics to gain physiological insight into why an individual’s arteries are stiff and identify new therapeutic strategies. In the Multi-Ethnic Study of Atherosclerosis, the important question of whether arteries stiffen with aging due to load-dependent or structural stiffening was investigated. Structural stiffening was consistently observed with aging, but load-dependent stiffening was highly variable. Importantly, the high load-dependent stiffness was associated with future CVD events, but structural stiffness was not. Clinical studies in older, hypertensive adults surprisingly show that decreasing VSMC tone can cause clinically significant increases in arterial stiffness. To understand this paradox, the author developed a model simple enough for clinical data but with biologically relevant ECM and VSMC parameters. The effect of VSMC tone on arterial stiffness depends on the ECM–VSMC stiffness ratio. Future research is needed to develop a framework that incorporates both the blood pressure dependence of arterial stiffness and the VSMC–ECM interaction on hemodynamics. This could result in personalized arterial stiffness treatments and improved CVD outcomes.

## Data Availability

Not applicable.

## Abbreviations

CVD: Cardiovascular disease
ECM: Extracellular matrix
MESA: Multi-Ethnic Study of Atherosclerosis
PWV: Pulse wave velocity
VSMC: Vascular smooth muscle cell
